# Transcriptomic Approach for Investigation of *Solanum* spp. Resistance upon Early-Stage Broomrape Parasitism

**DOI:** 10.3390/cimb46080535

**Published:** 2024-08-18

**Authors:** Maria Gerakari, Vasiliki Kotsira, Aliki Kapazoglou, Spyros Tastsoglou, Anastasios Katsileros, Demosthenis Chachalis, Artemis G. Hatzigeorgiou, Eleni Tani

**Affiliations:** 1 Laboratory of Plant Breeding and Biometry, Agricultural University of Athens, 11855 Athens, Greece; mgerakari@aua.gr (M.G.); katsileros@aua.gr (A.K.); 2 Hellenic Pasteur Institute, 11521 Athens, Greece; vasilikikotsira@hotmail.com (V.K.); spiros.tastsoglou@gmail.com (S.T.); 3 DIANA-Lab, Department of Computer Science and Biomedical Informatics, University of Thessaly, 35131 Lamia, Greece; 4 Hellenic Agricultural Organization-Dimitra (ELGO-DIMITRA), Department of Vitis, Institute of Olive Tree, Subtropical Crops and Viticulture (IOSV), Sofokli Venizelou 1, Lykovrysi, 14123 Athens, Greece; kapazoglou@elgo.gr; 5 Laboratory of Weed Science, Benaki Phytopathological Institute, 14561 Kifisia, Greece; d.chachalis@bpi.gr

**Keywords:** *Solanum* spp., broomrape, parasitism, transcriptomics

## Abstract

Tomato (*Solanum lycopersicum*) is a major horticultural crop of high economic importance. *Phelipanche* and *Orobanche* genera (broomrapes) are parasitic weeds, constituting biotic stressors that impact tomato production. Developing varieties with tolerance to broomrapes has become imperative for sustainable agriculture. *Solanum pennellii*, a wild relative of cultivated tomato, has been utilized as breeding material for *S*. *lycopersicum.* In the present study, it is the first time that an in-depth analysis has been conducted for these two specific introgression lines (ILs), IL6-2 and IL6-3 (*S*. *lycopersicum* X *S. pennellii*), which were employed to identify genes and metabolic pathways associated with resistance against broomrape. Comparative transcriptomic analysis revealed a multitude of differentially expressed genes (DEGs) in roots, especially in the resistant genotype IL6-3, several of which were validated by quantitative PCR. DEG and pathway enrichment analysis (PEA) revealed diverse molecular mechanisms that can potentially be implicated in the host’s defense response and the establishment of resistance. The identified DEGs were mostly up-regulated in response to broomrape parasitism and play crucial roles in various processes different from strigolactone regulation. Our findings indicate that, in addition to the essential role of strigolactone metabolism, multiple cellular processes may be involved in the tomato’s response to broomrapes. The insights gained from this study will enhance our understanding and facilitate molecular breeding methods regarding broomrape parasitism. Moreover, they will assist in developing sustainable strategies and providing alternative solutions for weed management in tomatoes and other agronomically important crops.

## 1. Introduction

Climate change impacts food security directly and indirectly. It imposes new limitations on resources essential to plant growth, affects crop–weed interactions, and exacerbates the negative effect of weed infestation on crop productivity, leading to severe losses in crop yield [1]. This can shift the balance of competition between host plants and parasitic weeds, potentially favoring the parasite over the host. Weeds comprise the major biotic factor limiting crop production worldwide [2]. Parasitic plants are scientifically intriguing and agriculturally important weeds that are spreading worldwide whereas at the same time, the means to control them remain limited [3]. Considering that parasitic weeds have evolved resistance towards some of the most widely applied herbicides, effective weed management has become an imperative for ensuring crop productivity [4]. The efficacy of classic herbicides has decreased due to their extensive use over the years [5], but they are not considered a sustainable agronomic practice.

The parasitic weed family *Orobanchaceae* (broomrapes) causes severe damage to economically important crops including legumes, sunflowers, and tomatoes [6]. They are widespread in Mediterranean areas, Asia, and Southern and Eastern Europe. Broomrape species attack dicotyledonous crops, depending entirely on their hosts for all nutritional requirements [7]. Due to their physiology, underground parasitism, achlorophyllous nature, and the hardly controlled seed bank, broomrapes cannot be effectively controlled by the usual management strategies designed for non-parasitic weeds [8]. Most of these strategies, such as preventive measures, cultivation practices, and biological and chemical control [9], fail to provide satisfactory control, as they are not feasible on a large scale, and often, they are not financially sustainable or environmentally safe [10,11]. One of these broomrape species namely *Phelipanche ramosa* is extremely widespread in the Mediterranean region and can parasitize many host crops of high nutritional and economic importance, including oilseed rape (*Brassica napus*), hemp (*Cannabis sativa*), tomato (*Solanum lycopersicum*), tobacco (*Nicotiana tabacum*), sunflower (*Helianthus annuus*), and melon (*Cucumis melo*) [12,13]. The recent state of the art regarding parasitic weeds–plant host interactions at the molecular level focuses on a newly discovered family of phytohormones, strigolactones (SLs), and their demonstrated involvement in host recognition and evolution of parasitic plants. Research by Cheng et al. [6] suggests that the development of low SL-producing lines in tomatoes may constitute a promising approach to combat the parasitic weed *Phelipanche ramosa*, as SLs are the main germination stimulants of this parasite. In this study, new findings have revealed that except SLs, there are also other metabolic pathways, and the respective underlying genes may be implicated in the complex interactions between host plants and parasitic weeds through the viewpoint of climate change.

Cultivated tomato is an important and very nutritional food resource for humans [14] and many commercial varieties are infected by broomrape species [15]. However, little is known about tomato defense mechanisms against broomrape parasitism, and despite extensive screening, no single immune strategy or strong resistance against broomrape has been reported [16]. Breeding for host resistance against holoparasitic weeds remains one of the most effective and sustainable management strategies to date, and considerable effort has been placed towards this goal for several crops such as peas and sunflower, over the last two decades [17]. Nevertheless, breeding for resistant varieties constitutes an ongoing struggle, as germplasm with broomrape-resistant traits remains scarce. Plant breeding programs based on molecular techniques seem to be the most promising and sustainable approach to effectively handle/manage the phenomenon of parasitism. A plethora of molecular and non-molecular techniques have been deployed in tomatoes aiming to increase the resistance of tomatoes to *Phelipanche* sp., such as the application of chemical or targeted mutagenesis and CRISPR/Cas9-mediated mutagenesis of specific targets as well as the use of appropriate rootstocks resistant to broomrape parasitism [18,19,20,21]. The vast genetic diversity found in the tomato’s wild relatives is associated often with resilience to different biotic and abiotic stress factors and adaptation mechanisms to extreme environmental conditions [22,23]. *Solanum pennellii*, a wild relative of the cultivated tomato, which is endemic to the Andean regions in South America, has evolved to thrive in arid habitats and has been used as breeding material for the cultivated *S. lycopersicum*, due to its stress tolerance to extreme conditions and unusual morphology [24]. *S. pennellii* ILs have been widely used to map QTLs influencing yield and fruit quality and are involved in heterosis [25]. ILs are also utilized as valuable genetic resources to improve abiotic stress tolerance [26].

RNA-Seq is probably the most widely applied high-throughput method to study gene expression at the transcript/isoform level. It involves the sequencing, quantification, and comparative analysis of the entire set of transcripts in an organism across tissues, developmental stages, and different conditions to assess the up- or down-regulation of genes involved in the relevant cellular processes [27]. PEA, also known as functional enrichment analysis (or pathway analysis), is typically applied to identify known molecular processes that are significantly enriched with genes that are altered in case samples in comparison to a control, facilitating interpretation and subsequent hypothesis generation [28].

Previous works employed the same approach to study the interaction of tomatoes with biotic stress factors. For example, RNA-sequencing and tissue-specific expression analyses were conducted to analyze the compatible interaction of tomatoes with *Verticillium dahliae* and *Cuscuta campestris*, respectively [29,30]. These studies aimed at developing novel sustainable breeding tools towards improved crop adaptability to environmental stresses that have been occurring with ever-increasing frequency and intensity over the last decades. Herein we provide, for the first time, a comparative transcriptomic and pathway analysis among putative tomato genotypes resistant and susceptible to the holoparasitic weed *P. ramosa*. Our study reveals differentially expressed genes in the host roots upon infection and metabolic pathways which may play a crucial role in the tomato response to parasitic infection and the establishment of resistance.

In the present study, a commercial tomato hybrid of *S. lycopersicum*, “Formula”, and two introgression lines (ILs) that were created by Eshed et al. [31] by crossing *S. lycopersicum* X *S. pennellii* (LA4060/IL6-2 and LA4062/IL6-3) were subjected to RNA-Seq and transcriptomic analysis to identify genes potentially implicated in tomato resistance against broomrape parasitism. In the present study, we did not use any synthetic bio-stimulants and we investigated the processes of parasitism and host response under natural conditions to simulate field environments and produce outcomes that could be practically useful to the farmers. IL6-2 and IL6-3 were selected based on previous work by Bai et al. [32] which showed resistance-associated QTLs residing in these lines. The analysis uncovered a large array of genes that were differentially expressed between parasitized and non-parasitized tomato plants in all three genotypes. Out of these, fourteen differentially expressed genes (DEGs) were further analyzed and validated using qPCR to reveal statistically significant differences in parasitized and non-parasitized samples within and between varieties. Selected genes were found to be implicated in specific aspects of host/parasite interactions such as the regulation of fatty acids and the role of several transcription factors which have been found to be involved in the regulation of plant secondary metabolism and their responses to environmental stressors [33,34,35]. PEA was also carried out revealing several key metabolic pathways to be involved in plants’ response against broomrape across the different tomato genotypes.

## 2. Materials and Methods

### 2.1. Plant Material and Treatment

**Figure 1:**
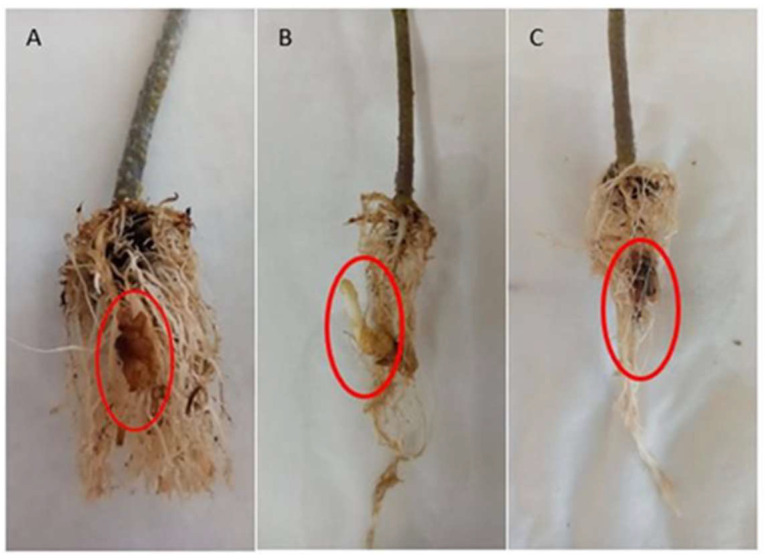
Parasitized tomato roots one month after inoculation with *O. ramosa* seeds. (**A**) Formula genotype parasitized with *O. ramosa* at the tubercule stage. (**B**) IL6-2 genotype parasitized with *O. ramosa* at the emergence stage. (**C**) IL6-3 genotype parasitized with *O. ramosa* at the early tubercule stage. Broomrape parasitism on tomato roots is presented in the red circles.

A well-known commercial tomato hybrid, ‘Formula’, with high susceptibility to broomrape parasitism as documented by the farmers and two introgression line (IL) populations (LA4060/IL6-2 and LA4062/IL6-3) developed by Eshed et al. [31] were selected for broomrape parasitism and molecular studies. These two specific ILs were selected based on the study of Bai et al. [32], as described. Each IL is homozygous for a single introgression from *S. pennellii* (LA0716) in the background of the cultivar (c.v) M82 (LA3475) of *S. lycopersicum* [31]. Fifty plants from each genotype were sown on disks under controlled temperature conditions (19–25 °C) in the greenhouse. When the fourth pair of true tomato leaves was visible (BBCH code 14, according to the tomato phenological growth stages key), forty plants were inoculated with *O. ramosa* seeds, while ten were maintained as non-parasitized control plants. Root infection observations began 30 days post-inoculation (Figure 1), and sampling was carried out at the flower spike development stage of broomrape. Tomato roots were collected both from control and parasitized plants, carefully removing the parasite from the roots of the host. Measurements of plant growth parameters (plant height and weight, soil plant analysis development (SPAD), and relative water content (RWC)) (Supplementary Tables S1, S2, S3, and S4, respectively) were recorded after roots’ inoculation with broomrape seeds. A one-way analysis of variance (ANOVA) was performed with six levels consisting of inoculated and non-inoculated plants of three genotypes.

### 2.2. Total RNA Extraction and Transcriptome Sequencing

Total RNA was extracted from tomato roots using the NucleoSpin RNA kit (MACHEREY-NAGEL, Dueren, Germany) according to the manufacturer’s instructions. Thermo Scientific NanoDrop ND-1000 Spectrophotometer UV/Vis was used to determine the concentration and quality of the RNA. Each sample’s concentrations ranged between 85 ng/uL and 260 ng/uL, and six biological replicates (three control plants and three parasitized plants) for each genotype were sequenced. Samples were subjected to cDNA library preparation (poly-A selection) and sequenced (150 nt paired-end reads) on an Illumina NovaSeq 6000 system (Novogene Europe, Cambridge, UK). On average 13.9 million reads were obtained per library (Supplementary File S2).

### 2.3. Transcriptomic Analysis

#### 2.3.1. Quality Control and Pre-Processing

Read quality was assessed with FastQCsuite [36]. In order to remove adapter sequences and trim low-quality reads from the raw sequencing data, the Cutadapt [37] tool was utilized, setting the minimum read length parameter at 30 and the quality threshold at 15.

#### 2.3.2. Genomic Alignment and Quantification

Pre-processed reads were subjected to splice-aware genomic alignment and quantification using STAR (v2.7.10) [38] and RSEM (v1.3.1) [39], respectively. Regarding sequencing libraries from *S. lycopersicum* samples (i.e., Fc1-3 and Fp1-3), a STAR genome index (STAR—runModegenomeGenerate) and an RSEM reference index (rsem-prepare-reference) were constructed using the GCF_000188115.5_SL3.1_genomic.fna genome assembly and providing the respective gene models (GCF_000188115.5_SL3.1_genomic.gtf). Genomic alignment (STAR—alignReads), allowing known and novel splice junctions and translating alignments into transcript coordinates (—quantModeTranscriptomeSAM), and quantification (rsem-calculate-expression) was performed, achieving a median 93.9% mapping rate. For sequencing libraries derived from introgression lines (i.e., IL6-2c1-3, IL6-2p2, IL6-2p3, IL6-2p5, IL6-3c1, IL6-3c2, IL6-3c4, and IL6-3p1-3), the mapping strategy inspired by the Szymański et al. [40] study was followed. Briefly, a combined STAR genomic index corresponding to both the *S. lycopersicum* (GCF_000188115.5_SL3.1_genomic.fna) and the *S. pennellii* (GCF_001406875.1_SPENNV200_genomic.fna) genomes was created. After splice-aware genomic alignment with STAR (median value of a 94.6% mapping rate), the alignment SAM records of each library were processed to create three discrete subsets:

(i) Slyc_subset, which contains multiple alignments (i.e., “NH” SAM tag > 1) on both *S. lycopersicum* and *S. pennellii* presenting the highest alignment score (“AS” tag in SAM) within *S. lycopersicum* and multiple alignments solely within *S. lycopersicum*, as well as unique alignments in *S. lycopersicum* (a median value of 85.2% of transcriptome-aligned reads across all datasets).
(ii) Penn_subset, which contains multiple alignments (i.e., “NH” SAM tag > 1) on both *S. lycopersicum* and *S. pennellii* presenting the highest alignment score within *S. pennellii* and multiple alignments solely within *S. pennellii*, as well as unique alignments in *S. pennellii* (a median value of 6.6% of transcriptome-aligned reads across all datasets).
(iii) Ambi_subset, which contains multiple alignments on both *S. lycopersicum* and *S. pennellii* presenting equally high alignment scores (i.e., ties) in both species (a median value of 8.1% of transcriptome-aligned reads across all datasets). Entries on *S. pennellii* were retained for these ambiguous alignments for downstream analysis.

RSEM (rsem-calculate-expression), provided with a combined reference index containing gene models of both species, was used on each set separately to produce read count estimates. Supplementary Tables S5 and S6 present alignment and quantification metrics for all generated datasets.

### 2.4. Differential Expression Analysis

As previously described, the analysis proceeded following the strategy outlined by Szymański et al. [40]. The subset of alignments in both genomes (Ambi subset) was generated for *S. pennellii*. Differential expression analysis was performed using the programming language R (v4.2.2; R Core Team 2021) and the R package edgeR (version 3.40.2) [41]. Specifically, the differential expression analysis for each line was conducted twice: once for the genes aligned in the *S. pennellii* genome and again for the genes aligned in the *S. lycopersicum* genome. Low-abundance genes were filtered by employing the filterByExpr function with default arguments. The weighted trimmed mean of the M-values (TMM) normalization method and quasi-likelihood F-testing were applied to assess abundance change in the following pair-wise contrasts: (i) Fc vs. Fp (Formula); (ii) IL6-2c vs. IL6-2p (*S. pennellii* genes); (iii) IL6-2c vs. IL6-2p (*S. lycopersicum* genes); (iv) IL6-3c vs. IL6-3p (*S. pennellii* genes); and (v) IL6-3c vs. IL6-3p (*S. lycopersicum* genes). The false discovery rate (FDR) was controlled using the Benjamini–Hochberg procedure [42], and the statistical significance threshold was set at *p* adjusted to <0.05.

### 2.5. Functional Enrichment Analysis

The limma R package (version 3.54.2) [43] was utilized for pathway enrichment analysis, employing the kegga function with default parameters and the KEGG database. The differentially expressed genes were subjected to pathway analysis, applying two different approaches due to the absence of a standardized method in the literature. In the first approach, separate analyses were conducted for each of the species of each introgression line, having the genes originating from *S. lycopersicum* considered separately from the genes derived from *S. pennellii*. In the second approach, since the number of pathways and their names were the same for the two different species of each introgression line, genes from both were merged into a single gene set within each pathway, resulting in a single pathway analysis for each introgression line. Each of these approaches has its own advantages and limitations. In the first scenario, the study is analogous to examining two distinct organisms, potentially leading to information loss within the hybrid formula. In the second instance, the presence of duplicates in pathways is highly probable, due to the inability to identify corresponding genes between the two species owing to discrepancies in gene IDs and names within the annotation files, possibly impacting the subsequent statistical analysis. A pathway was deemed significant if its corresponding adjusted *p*-value (Benjamini–Hochberg correction) was below the threshold of 0.05.

### 2.6. Visualizations

Visualizations were generated using the following R packages for each task: volcano plots were generated using ggplot2 (version 3.4.4) [44], bar plots were created using both ggplot2 and ggpubr (version 0.6.0) [45], heatmaps were generated using limma (version 3.54.2), gplots (version 3.1.3.1) [46], and gridExtra (version 2.3) [47], and Venn diagrams were created using VennDiagram (version 1.7.3) [48].

### 2.7. Gene Expression Analysis Validation by qRT-PCR

Quantitative Reverse Transcription–PCR (qRT-PCR) was performed to verify the RNA-Seq results. Total RNA was extracted from the three genotypes (Formula, IL6-2, and IL6-3) and used for RNA-Seq analysis, as described above. First-strand cDNA was synthesized from 500 ng of total RNA using the PrimeScript™ 1st strand cDNA Synthesis kit (Takara Bio Inc. Lab Supplies P.GALANIS & CO, Athens, Greece). Gene-specific primers for selected DEGs were designed using the NCBI Primer-Blast tool (https://www.ncbi.nlm.nih.gov/tools/primer-blast/, accessed on 30 January 2023). The list of the DEGs selected for qPCR validation and their data after bioinformatic analysis are presented in Supplementary Table S7. Primers and GenBank accession numbers of genes are listed in Supplementary Table S8. qPCR was performed in a total volume of 20 μL using the PowerUpTM SYBRTM Green Master Mix (Applied Biosystems) and was conducted in a StepOnePlusTM Real-Time PCR System machine (Thermo Fisher Scientific, Waltham, MA, USA). PCR conditions were applied according to the manufacturers’ instructions. Each reaction was performed in three technical triplicates along with the internal control reaction. Gene expression levels were determined with StepOnePlusTM Real-Time PCR manual 2-delta-delta-Ct method [49], and R programming language (v4.2.2; R Core Team 2021) software was applied for the statistical analysis. Three biological repeats were used for the experiment.

## 3. Results

### 3.1. Plant Growth

Measurements regarding height, weight, and soil plant analysis development (SPAD) were recorded on four different dates (Supplementary Tables S1–S3), while one RWC measurement was carried out too (Supplementary Table S4). The results did not indicate any significant differences for weight and RWC measurements. Regarding the comparison of plant height between non-inoculated and inoculated plants, on average, inoculated plants were taller. SPAD measurements presented differences only in the third week after inoculation when Formula appeared to be the most affected genotype with decreased SPAD values. Inoculated plants gave better SPAD values than non-inoculated plants for the three genotypes on all other dates. The above results agree with a previous report, demonstrating that host plants begin to be significantly affected approximately 40 days post-infection, highlighting the fact that early stages of parasitism do not elicit profound morphological/physiological differences across genotypes [50]. Furthermore, during the experimental procedures, visual observations were taking place regarding the percentage of parasitism in the infected plants of all genotypes. Forty plants were inoculated with broomrape seeds in total for the three genotypes studied. We observed 80% parasitism at Formula roots, while IL6-2 and IL6-3 had 45% and 35% parasitized roots, respectively.

### 3.2. Comparative Transcriptomic Analysis Reveals Differentially Regulated Genes upon Parasitism

**Figure 2:**
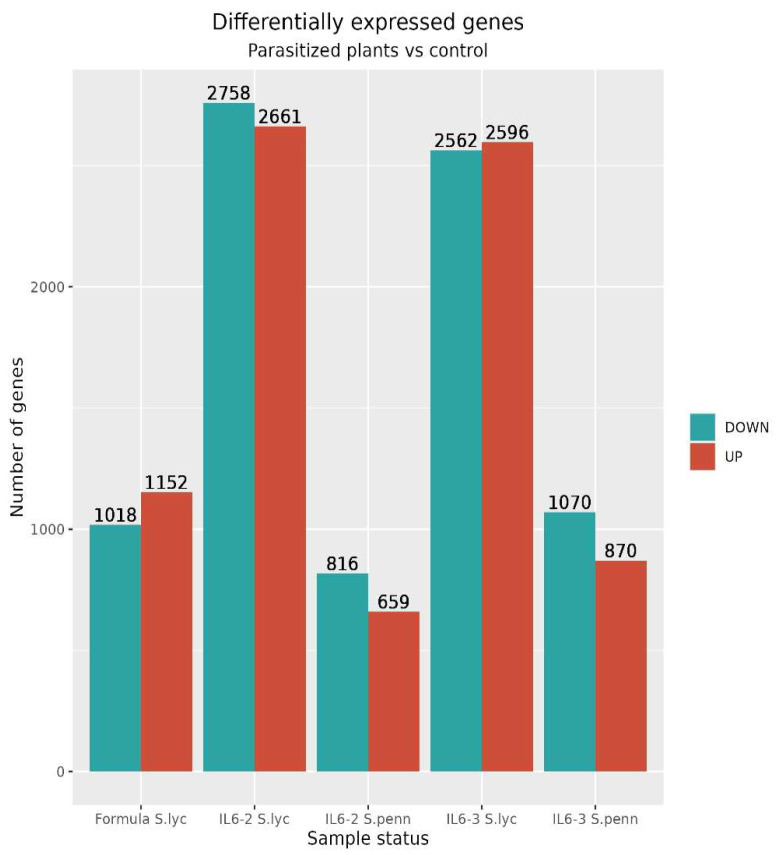
Bar plot illustrating numbers of significant DEGs (FDR < 0.05) for the Formula genotype and the two introgression lines, based on the results obtained from edgeR. Each line produced two sets of results since differential expression analysis was conducted once for genes originating from the *S. pennellii* genome and the second for genes originating from the *S. lycopersicum* genome. Down-regulated genes in the presence of *Phelipanche* are represented in the blue color, while up-regulated ones are shown in red.

**Figure 3:**
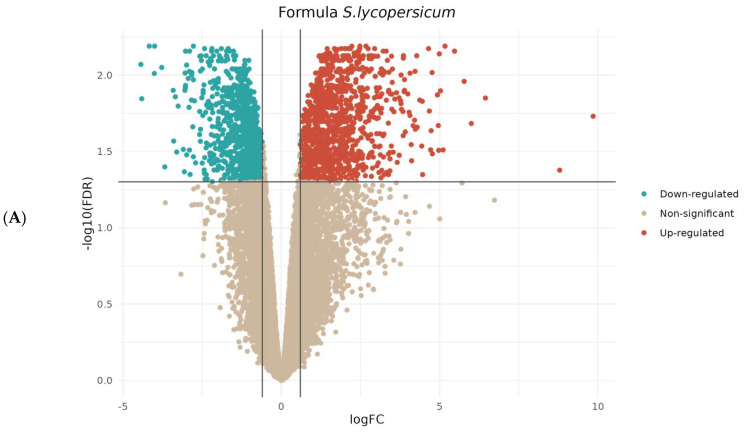

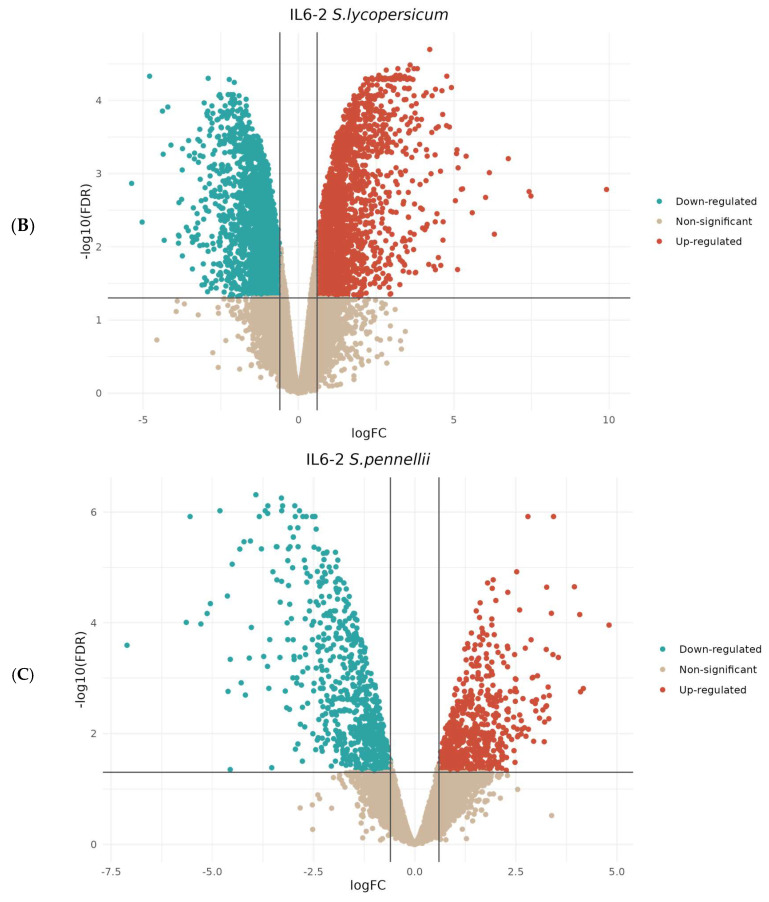

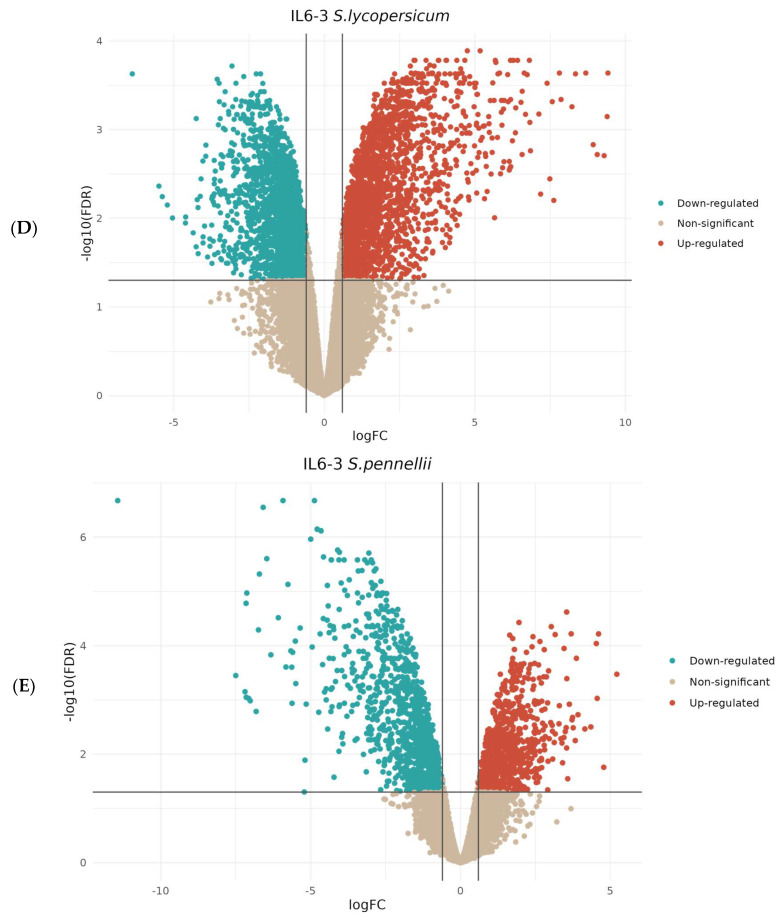
Volcano plots demonstrating results of differential expression analysis between parasitized and non-parasitized plant samples of (**A**) ‘Formula’ with *S. lycopersicum* annotated genome, (**B**) IL6-2 with *S. lycopersicum* annotated genome, (**C**) IL6-2 with *S. pennellii* annotated genome, (**D**) IL6-3 with *S. lycopersicum* annotated genome, and (**E**) IL6-3 with *S. pennellii* annotated genome. Genes are represented as points and are colored if |log_2_(fold change)| > 0.6 and adjusted to *p* < 0.05. Up-regulated genes are highlighted in red, while down-regulated genes are highlighted in blue color.

**Figure 4:**
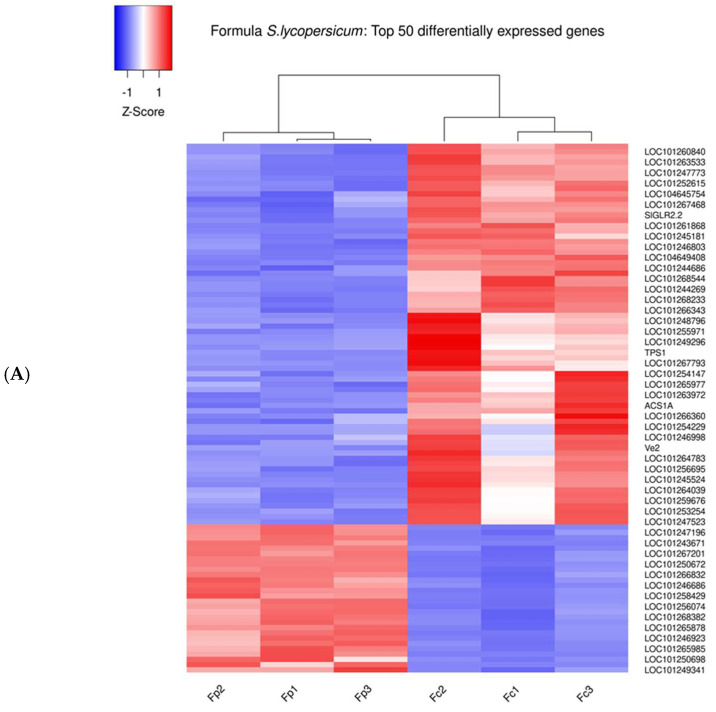

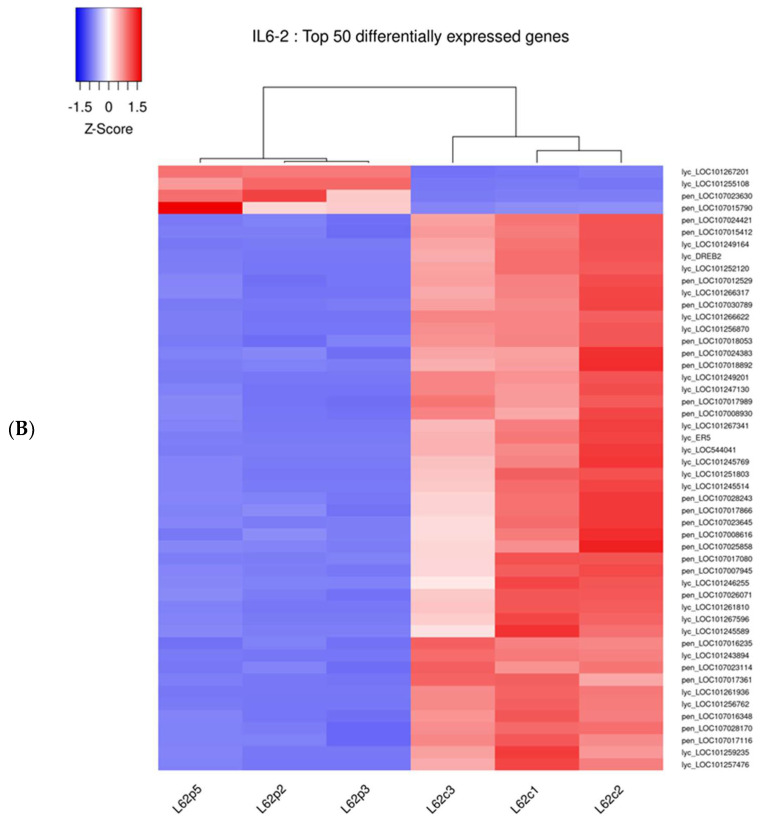

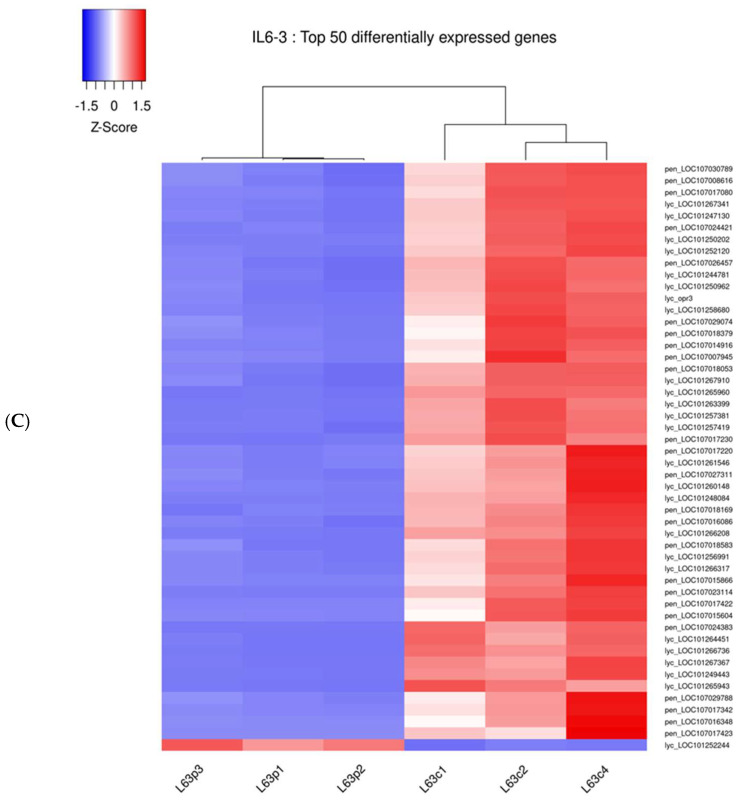
(**A**) Top 50 DEGs (up- and down-regulated) in Formula genotype. Fp1, Fp2, and Fp3 correspond to parasitized plants of Formula hybrid, while Fc1, Fc2, and Fc3 refer to Formula controls (i.e., non-infected roots). (**B**) Top 50 DEGs (up- and down-regulated) in IL6-2 genotype. IL6-2p2, IL6-2p3, and IL6-2p5 correspond to parasitized plants of IL6-2, while IL6-2c1, IL6-2c2, and IL6-2c3 refer to IL6-2 controls. (**C**) Top 50 DEGs (up- and down-regulated) in IL6-3 genotype. IL6-3p1, IL6-3p2, and IL6-3p3 correspond to parasitized plants of IL6-3, while IL6-3c1, IL6-3c2, and IL6-3c4 refer to IL6-3 controls. Color-coding represents z-score-scaled normalized read counts for each gene across samples.

During the sampling day, phenotypic observations on roots regarding the number of parasitized plants revealed that the IL6-3 line was less infected compared to the other two genotypes, while Formula had the most infected roots. These observations confirmed the initial hypothesis that the commercial hybrid is possibly more susceptible compared to the introgression lines. The transcriptomic analysis results unveiled a significant number of DEGs, displaying both up- and down-regulation across most of the tested genotypes (Figure 2 and Supplementary File S2). The analysis for each genotype involved comparing the parasitized samples with their respective controls. The examination of two introgression lines (ILs) was conducted separately for genes originating from *S. pennellii* and *S. lycopersicum*. Thus, for each line, two distinct comparisons were performed. The results are visually presented in the volcano plots below (Figure 3B,C for IL6-2 and Figure 3D,E for IL6-3). Differential expression analysis revealed a notable number of DEGs in all genotypes at the parasitized plants with a very clear distinction between up-regulated and down-regulated genes. Moreover, it should be emphasized that in both ILs, a considerable number of up- and down-regulated genes originated from the *S. pennellii* genome (i.e., 1475 and 1940 for IL6-2 and IL6-3, respectively). Additionally, as depicted in Figure 2, the number of significant genes for *S. lycopersicum* was 5428 and 5158 for IL6-2 and IL6-3, respectively. Heatmap depictions indicated differences in gene expression comparing parasitized and non-parasitized tomato roots of the three studied genotypes. In each heatmap, the top 50 DEGs are presented for each genotype (three control samples and three parasitized samples per genotype). In Formula’s parasitized roots (Fp), 14 out of 50 DEGs show up-regulation, while the same 14 genes are down-regulated for the controls of Formula (Fc) (Figure 4A). In IL6-2, we observe that genes’ up-regulation is more targeted in parasitized plants (IL6-2p) compared to controls (IL6-2c) as there are 4 out of 50 up-regulated genes (2 originated from *S. lycopersicum* and 2 from *S. pennellii*). The rest of the DEGs are down-regulated in parasitized plants, while in control samples, the same genes are up-regulated (Figure 4B). Regarding IL6-3, the vast majority of DEGs are down-regulated in parasitized plants (IL6-3p), while in control samples, the same genes are up-regulated (Figure 4C). All 50 genes in the two ILs originate from both *S. lycopersicum* and *S. pennellii*. From these results, we can assume that ILs’ resistance mechanisms against broomrape parasitism are more targeted which is presented by the up-regulation of very few genes.

### 3.3. The 14 DEGs in Response to Broomrape Parasitism

Following transcriptomic analysis, we opted to select key candidate genes for validation and further study. Based on DEGs and the existing literature, fourteen genes were subjected to qPCR analysis. The genes’ selection for validation was based on the bioinformatic analysis results of the RNA-Seq (Supplementary File S2, Supplementary Table S7) as well as on previous research studies. Table 1 elucidates the description of the studied genes in the NCBI and SolGenomics databases and the gene names in the current study.

### 3.4. qPCR Validation Highlights Prominent Changes upon Parasitism in IL6-3 Line

**Figure 5:**
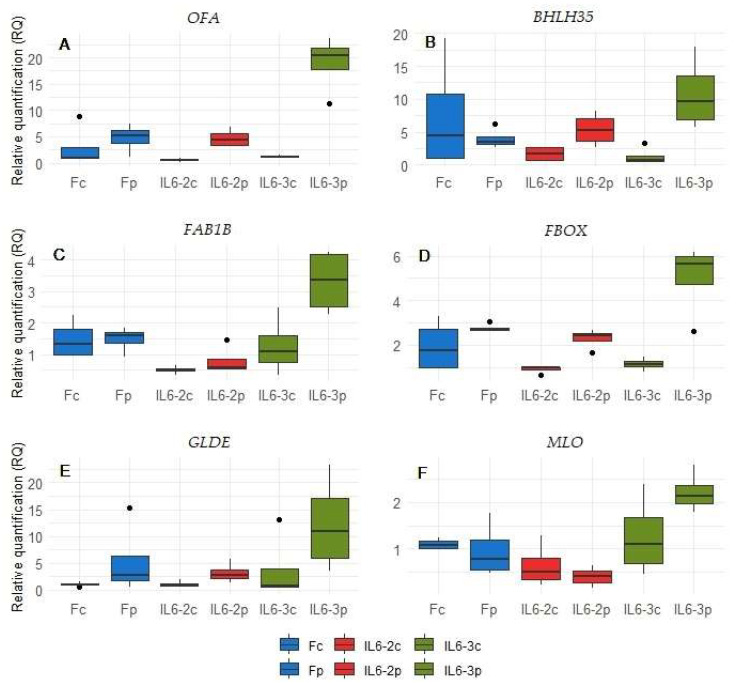
Relative expression levels per genotype and treatment (control vs. parasitized plants) of 6 genes denoted as DEGs in the RNA-Seq experiments, which appear as significantly up-regulated in IL6-3 parasitized plants via qPCR. Treatments: Formula control (Fc), Formula parasitized (Fp), introgression line 6-2 control (IL6-2c) and parasitized (IL6-2p), and introgression line 6-3 control (IL6-3c) and parasitized (IL6-3p). Statistically significant differences were determined at *p* < 0.05 comparing the relative expression between the control and the parasitized plants/genotype.

Relative expression levels were normalized with Actin (ACT) gene expression levels, and statistically significant differences were determined at a 95% confidence interval. All genes were expressed in the genotypes under study; however, statistically significant differences were observed for 9 out of 14 candidate genes, especially for the genotype IL6-3. With respect to *OFA*, *BHLH35*, *FAB1B*, *FBOX*, *GLDE*, *MLO*, *ZINC*, *PPDG*, and *CaM2* DEGs, the relative expression levels with statistically significant differences were observed in parasitized plants, mostly in IL6-3, validating the transcriptomic analysis results. In particular, *OFA*, *BHLH35*, *FAB1B*, *FBOX*, *GLDE*, and *MLO* DEGs displayed significant up-regulation in the parasitized plant of IL6-3 (Figure 5).

Specifically, in parasitized IL6-3, *OFA* and *BHLH35* exhibited a significant ~18- and 11-fold increase in gene expression, respectively, as compared to the control samples (Figure 5A,B). Similarly, *FAB1B* and *FBOX* displayed a significant but lower up-regulation of 3- and 5-fold, respectively, compared to their controls (Figure 5C,D). Finally, a 4- and 2-fold increase was evidenced for *GLDE* and *MLO*, respectively, in parasitized IL6-3 (Figure 5E,F).

**Figure 6:**
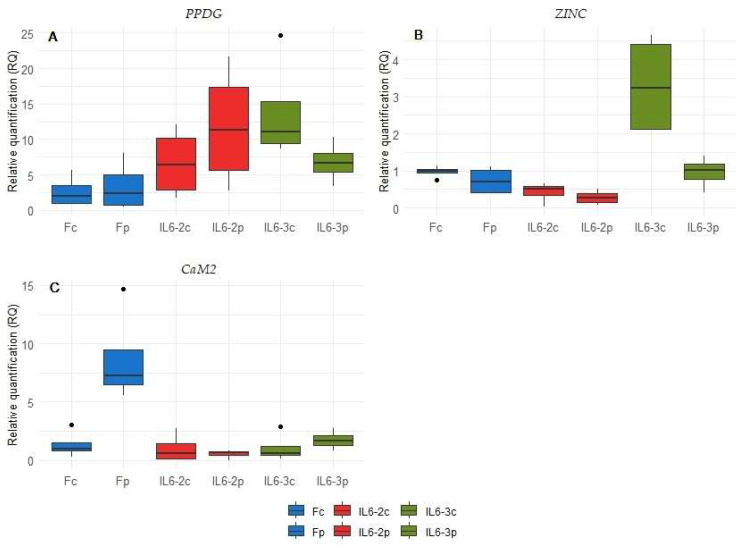
Relative expression levels per genotype and treatment (control vs. parasitized plants) of 3 genes denoted as DEGs in the RNA-Seq experiments, which appear as significantly up-regulated in IL6-3 control plants compared to parasitized ones ((**A**,**B**), *PPDG*, and *ZINC*, respectively) and up-regulated in Formula parasitized plants ((**C**), *CaM2*). Treatments: Formula control (Fc), Formula parasitized (Fp), introgression line 6-2 control (IL6-2c) and parasitized (IL6-2p), and introgression line 6-3 control (IL6-3c) and parasitized (IL6-3p). Statistically significant differences were determined at *p* < 0.05 comparing the relative expression between the control and the parasitized plants/genotype.

Significant down-regulation for the parasitized plants of IL6-3 was also observed among the fourteen selected genes. In particular, *PPDG* and *ZINC* displayed a 2- and 3-fold decrease in parasitized IL6-3, respectively (Figure 6A,B). *CaM2* gene discriminated its relevant expression only in parasitized plants of Formula, where an 8-fold change was observed compared to the control plants (Figure 6C).

*ACLA2*, *D14*, *GABA*, *PSY*, and *ZPR1* genes were additionally selected for qPCR analysis based on previous publications regarding transcriptional responses to biotic stressors. They were expressed in all tested genotypes in both parasitic and control plants; however, no statistically significant differences were revealed in their relative gene expression (Supplementary Figure S1).

### 3.5. KEGG Enrichment Analysis of DEGs

**Figure 7:**
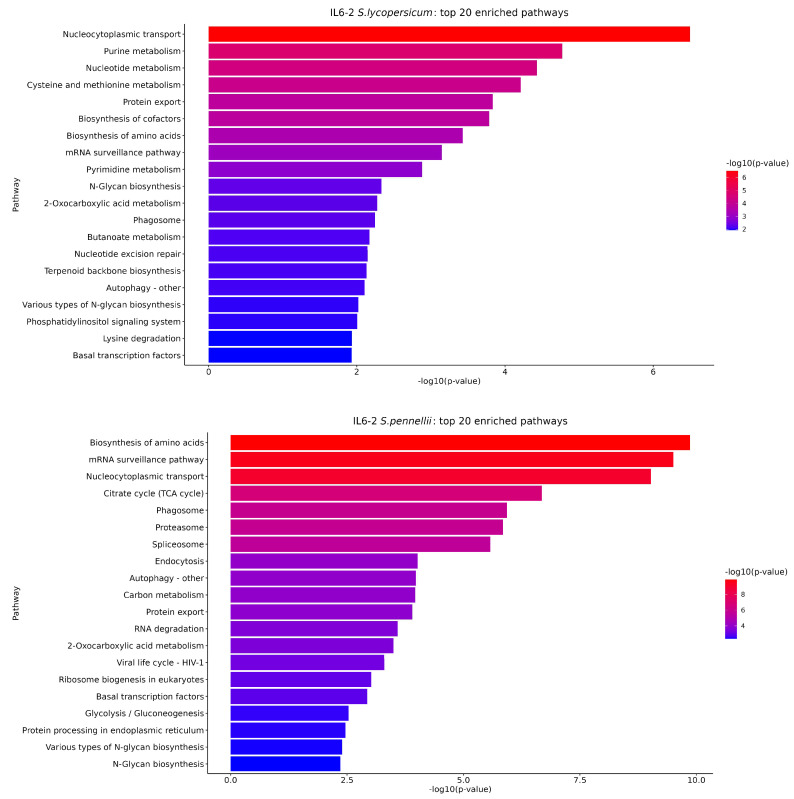
The top 20 significantly enriched pathways, obtained from the first approach pathway analysis for introgression line IL6-2, with *p* < 0.05.

**Figure 8:**
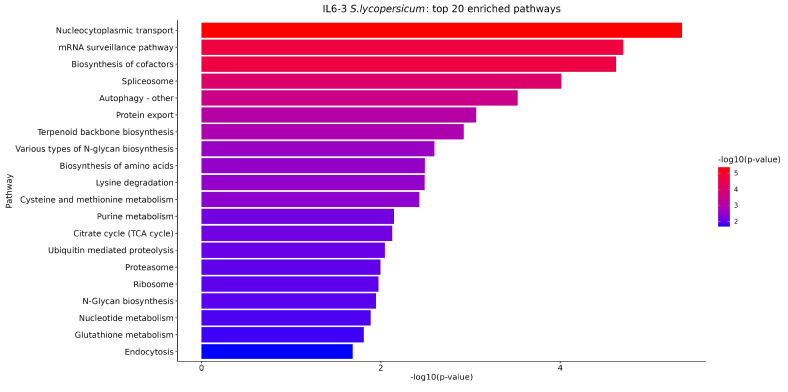

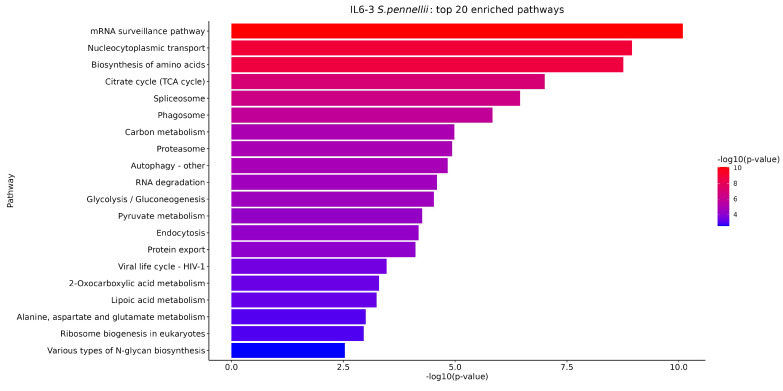
The top 20 significantly enriched pathways, obtained from the first approach pathway analysis for introgression line IL6-3, with *p* < 0.05.

**Figure 9:**
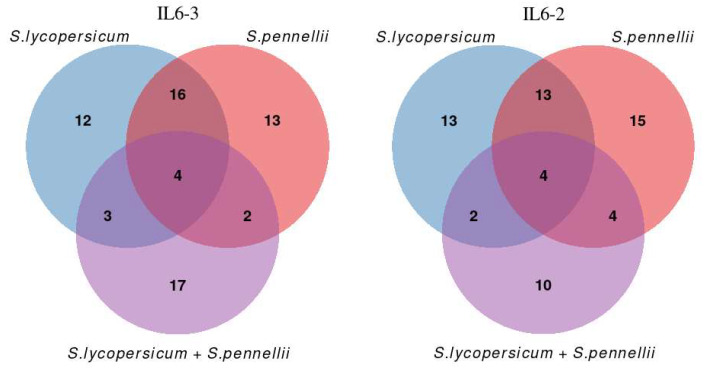
Venn diagram illustrating numbers of common pathways identified as significant across different genomic annotations.

PEA of the RNA-Seq findings was carried out with two distinct approaches. It is worth noting that approximately 11,000 genes in both species (out of ~31,000 total) are annotated with regard to biological pathways in the Kyoto Encyclopedia of Genes and Genomes (KEGG) resource [51], and thus used as background for testing. Regarding the first approach, we analyzed the genes of each species separately in each introgression line, and the top 20 significantly enriched pathways are presented in Figure 7 and Figure 8. Specifically, the number of significantly enriched pathways for IL6-2 was 32 regarding *S. lycopersicum* and 36 regarding *S. pennellii*, respectively. For line IL6-3, the number of significantly enhanced pathways was 35 regarding both *S. lycopersicum* and *S. pennellii*. For the second approach, in which genes of the two species that belong to the same pathway were merged into a single set, the results can be found in Supplementary Figure S2. Additionally, a Venn diagram was generated to visualize the number of significantly enriched pathways found to be shared/unique among the two different species in each IL (Figure 9).

Notably, out of the 14 genes subjected to qPCR validation, only 4 are currently included in pathway annotations provided by KEGG. Out of these, 3 pertain to metabolic and signaling pathways that were found to be significantly enriched by DEGs in our transcriptomic analyses (Table 2).

## 4. Discussion

Managing broomrapes effectively requires a combination of strategies. The difficulties associated with their control, such as host specificity, seed bank persistence, underground growth, etc., are well documented (and reported in the international bibliography) [6,52]. However, the efficacy of these strategies in combating the parasite remains very constrained. Notably, timely prevention of root parasitism prior to attachment of the parasite to the root is of crucial importance [8]. The present study aimed to unravel significant differences in the expression of key genes during the early stage of parasitic infection, without utilizing stimulants, but instead by mimicking field conditions of parasitism. Differentially expressed key genes will be further investigated towards developing appropriate marker tools for the identification of genetic material with broomrape tolerance. Notably, several transcriptomic studies regarding tolerance to *Orobanche* have investigated the molecular response to infection at a single time point [53,54], which resembles our approach.

RNA-Seq analyses of host plants upon parasitism can provide a high-throughput view of the alterations induced as they interact with a parasite, including changes in gene expression, biochemical pathways, and host defense regulatory elements. Key parameters that are of particular interest include resistance genes, defense signaling pathways, hormonal regulatory components, and secondary metabolites implicated in the response to the parasite infection and development of resistance [55,56]. A plethora of transcriptomic analyses has been carried out in the past in tomato cultivars parasitized with broomrape species [56,57,58].

Our study is the first to perform an in-depth analysis of two specific ILs (IL6-2 and IL6-3) upon infection with the holoparasite and to unravel important DEGs originating from the *S. lycopersicum* and/or the *S. pennellii* genome. The aim was to validate several DEGs derived from transcriptomic analysis and relate them with significant pathways identified from each species (*S. lycopersicum* and *S. pennellii*). The overwhelming majority of DEGs revealed in this study are related to cellular and metabolic processes, responses to stimuli, and the regulation of protein synthesis. Prior studies on parasite–host interactions and crop resistance have focused on genes related to the metabolic pathway of strigolactones, a group of terpenoid lactones derived from carotenoids [3]. Fernández-Aparicio et al. [59] carried out research in fava beans, demonstrating that low strigolactone exudation within fava bean germplasm contributes to broomrape resistance. A recent review discusses different germination stimulants and their potential role in host specificity [60]. While very promising, strigolactone’s regulation is not the only approach to manipulate broomrape resistance in crops. A recent transcriptomic study has demonstrated the significant contribution of the salicylic acid (SA) pathway on sunflower resistance to *Phelipanche cumana* during parasitism [61].

Our transcriptomic analysis revealed DEGs between parasitized and control plants, the vast majority of which (a) were found up-regulated in response to broomrape parasitism and (b) have crucial roles in processes other than strigolactone regulation. Omega-3 fatty acid desaturases (*FADs*) are enzymes that catalyze the conversion of omega-3 fatty acids from their precursor omega-6 fatty acids. In plants, *FADs* are important for maintaining membrane fluidity and stability, being key factors contributing to tolerance to various environmental stresses [62,63]. The studied FAD family member (named *OFA* gene throughout the manuscript) exhibited a 20-fold increase in parasitized roots of IL6-3 compared to the control and remained at control levels in the other two genotypes under study. The results by Shi et al. [63] associate an increased expression of *CbFAD3* in tobacco to tolerance against multiple stressors. Regarding the *FAB* gene family, *FAB1A* and *FAB1B* genes in *Arabidopsis* are genes that are important for the maintenance of endomembrane homeostasis [64]. The *FAB1B* gene, known for its role in regulating plant growth and stress responses, encodes a phosphatidylinositol-3-phosphate 5-kinase, an enzyme involved in the regulation of membrane homeostasis, trafficking, and cellular signaling [64]. Both genes, *FAD* (*OFA*) and *FAB1B* were found overexpressed in the parasitized roots of IL6-3, indicating/suggesting potential functional roles under biotic stress conditions, such as broomrape infestation. This constitutes the first research work implicating a role for *FAB* genes in broomrape tolerance, while very recently published data link *FAD-like* genes to broomrape resistance [65]. These findings highlight (*OFA*) and *FAB1B* as promising candidates for further study towards a better understanding of the defense mechanisms of tomato cultivars against broomrape infestation.

The basic helix–loop–helix (BHLH) family of transcription factors is extensively studied and has been implicated in the regulation of plant secondary metabolism [66], associated with responses to environmental stressors [67]. Jiang et al. [68] have reported that heterologous overexpression of the *Anthuriuman draeanum BHLH35* gene (*AaBHLH35*) in *Arabidopsis* transgenic lines enhanced tolerance to cold and drought stresses, supporting the notion that *BHLH35* is an essential factor for abiotic stress tolerance. Considering the findings of studies linking increased *BHLH* expression with enhanced tolerance to *Phelipanche* species [67], we hypothesize that *BHLH35* could be another promising candidate gene for conferring partial resistance to *P. ramosa* in IL6-3.

The *F-box* gene family is one of the most abundant and pleiotropic families in plants [69,70], with members that can be either positive or negative regulators of hormone biosynthesis [71,72,73]. The involvement of the F-box protein *MAX2* in strigolactone biosynthesis has been demonstrated [74], and further structural and functional analysis of our *F-box* gene can elucidate its correlation to strigolactone biosynthesis and its specific role in broomrape tolerance.

The *MLO* gene encodes a negative regulator of defense responses in plants, which has been reported as a key regulator of resistance to powdery mildew in tomatoes [75]. In general *MLO* gene appears to be important in regulating plant defense responses to biotic stress factors [76].

The *glutamate decarboxylase 4* gene *GAD* (named *GLDE* gene throughout the manuscript) encodes the glutamate decarboxylase 4 (GAD4) protein. In plants, *GAD* genes are crucial in regulating GABA levels, which in turn affects plant growth and development, as well as the response to environmental stresses [77]. Calmodulin (CaM) is a calcium-binding protein that also controls various cellular processes, including signal transduction, gene expression, and stress responses. Plants employ the divalent cation calcium (Ca^2+^) as a second messenger in relaying these endogenous (developmental) and exogenous (environmental) signals to appropriate cellular responses [78]. Very few studies have reported the implication of both genes to stress tolerance. Rajani et al. [79] demonstrated that maize plants expressing the deregulated *AtGAD1* exhibit severe chlorosis, retarded growth phenotype, high levels of GABA, and Ca^2+^/CaM-independent *GAD* activity, indicating that both genes are involved in the response of plants to stress factors. These findings agree with the results of the present study, where *GLDE* and *CaM2* are found up-regulated in broomrape-infected plants of IL6-3 and Formula genotypes, respectively.

Phospholipase D (PLD) enzymes cleave phospholipids to generate the lipid second messenger, phosphatidic acid (PA). In *Arabidopsis thaliana*, *PLDγ1-like* (named *PPDG* gene throughout the manuscript) is expressed in various plant tissues and is involved in the regulation of plant responses to abiotic and biotic stresses [80]. Zinc finger CCCH domain-containing proteins (ZFPs) are a subfamily of zinc finger proteins that contain a conserved CCCH motif. Tomatoes feature several *ZFPs-encoding* genes, including the Zinc finger CCCH domain-containing protein 32 (SlZFP32), a transcription factor involved in regulating genes implicated in abiotic stress responses [81]. *PPDG* and *ZINC* genes are down-regulated in parasitized plants compared to the controls, especially in IL6-3, revealing an interesting pathway of potential tolerance for further study.

Additionally, three other genes *CaM2*, *PPDG*, and *ZINC* displayed down-regulation in parasitized IL6-3 roots, an expected outcome based on their gene ontology description. More specifically, based on the description of gene ontology data at the Sol genomics database, the *CaM2* gene (GO:0075135) is implicated in processes in which the symbiont organism (the host organism—tomato) stops, prevents, or reduces the frequency, rate, or extent of calcium or calmodulin-mediated signal transduction. Therefore, it could be suggested that *CaM2* should be suppressed in the tolerant host genotypes inoculated with broomrapes. Formulas’ parasitized plants exhibited a marked increase in *CaM2* expression, as compared to control plants as well as to the other two ILs, which supports the notion of Formula’s susceptibility to the parasite.

Moreover, the *PPDG* gene studied in this work has been found to be a negative regulator in various biotic and abiotic stressors. More specifically, according to the gene ontology description, *PPDG* presents negative regulation of the response to biotic stimulus (GO: 0002832) like stringolactones (SLs), a major mechanism involved in broomrape germination. Furthermore, it provokes negative regulation of filamentous growth of a population of unicellular organisms in response to a chemical stimulus (GO: 1900438) (filamentous growth also inoculates the hosts’ roots, and the hosts use similar resistant mechanisms to avoid parasitism like in broomrapes). It also appears as a negative regulator of defense response (GO:0031348). These are only a few examples of *PPDG*’s negative regulation of plant tolerance to biotic stressors. These results agree with the findings of this study, where *PPDG* is down-regulated in IL6-3 parasitized plants, further supporting our suggestion that this specific introgression line is the most tolerant of the three genotypes tested.

Regarding the *ZINC* gene, and based on the gene ontology data, it codes for a host cell extracellular matrix binding protein, interacting selectively and non-covalently with the extracellular matrix of a host cell (GO:0046810). In our study, *ZINC* displayed up-regulation in control roots (non-parasitized with broomrape) in IL6-3, compared to all the other genotypes. It might serve as a repressor of transcription whose down-regulation in parasitized IL6-3 roots allows for downstream stress-responsive genes to be transcribed. In conclusion, the three down-regulated genes found in this research are all in accordance with the gene ontology data of the Solgenomics database, which is the most specific, updated, and trustworthy database for Solanaceae plants. Based on these findings, these genes could be further studied to demonstrate a putative role in tomato resistance against *Phelipanche*. Our results are in line with existing reports regarding the involvement of these genes in abiotic/biotic stressors and imply possible roles in broomrape resistance. The differential gene expression could possibly be due to the *S. pennellii* genome or to the complementary interaction of both genomes. Future studies assessing the precise genomic content of these lines, as well as their epigenomic activity under parasitism, could shed more light on the exact mechanisms. Partial resistance against a different broomrape species, *Phelipanche aegyptiaca*, was also found by Bai et al. [75] in these ILs.

PEA identifies biological terms that are overrepresented in a group of genes more than would be expected by chance and ranks these terms by significance [82]. Dai et al. [83] carried out a comparative transcriptome analysis between the cultivated and wild tomato genotypes under abiotic stressors (i.e., salt and drought stress). Their enrichment results using the KEGG resource revealed numerous implicated pathways of interest, some of which are also found through our analysis, including the biosynthesis of amino acids, carbon metabolism, glycolysis/glyconeogenesis, and the ribosome pathway (Figure 6 and Figure 7). A de novo comparative transcriptome analysis of DEGs in the scion of homografted and heterografted tomato seedlings carried out by Wang et al. [84] presented the 10 most significantly enriched KEGG pathways among DEGs detected between hetero- and homografted tomato seedlings. Grafting might cause abiotic stress effects to the seedlings, and their analysis pinpointed pathways like our study, such as the MARK-signaling pathway plant, plant hormone signal transduction (Supplementary Figure S2), the biosynthesis of amino acids, protein processing in the endoplasmic reticulum, the spliceosome, etc. (Figure 6 and Figure 7). Furthermore, Ashapkin et al. [85], in a review study regarding genomic and epigenomic mechanisms of host plants against the parasitic weed *Cuscuta*, mentioned that *Cuscuta japonica* exhibited distinct morphological features after attachment to *Ficus macrocarpa* (host plant) and *Mangifera indica* (non-host plant). A KEGG PEA was carried out, revealing various important biochemical pathways, significantly enriched by DEGs. Several pathways are also demonstrated in our study (Figure 7, Supplementary Figure S2), such as “flavonoid biosynthesis”, “plant–pathogen interaction”, “arginine and proline metabolism”, “MARK signaling”, “phenylalanine metabolism”, “arginine biosynthesis”, and “carbon fixation in photosynthetic organisms”. The results presented by Ashapkin et al. [85] indicated that many metabolites and signal pathways are responsible for rendering host resistance to dodders. To determine *Striga* germination rates, one of the most serious parasitic weeds, infesting various *Sorghum* genotypes, Irafasha et al. [86] categorized their samples by hierarchical clustering using self-organizing maps (SOMs). The PEA of SOMs of DEGs revealed various pathways that control several important biological regulations such as seed dormancy break and germination. Specific gene expression patterns in the ABA biosynthesis pathway are also mapped in the carotenoid biosynthesis pathway involving enzymes like PSY, which is also involved in our study. Furthermore, it is also referred that the observations reveal possible interactions between SL, ABA, and other hormone pathways [86]. These similarities indicate that plant resistance mechanisms against biotic and abiotic stressors could, to some extent, exhibit shared mechanistic components and interactions.

Ten out of fourteen genes assessed via qPCR in our study do not appear to be involved in significantly enriched pathways. We should emphasize, however, that 11,000 out of 31,221 total genes in *S. lycopersicum* (35%) are currently annotated to KEGG pathways. This underscores the fact that our knowledge of the genes’ roles in molecular mechanisms is still incomplete, and this phenomenon is exacerbated in plant organisms. The validated genes of unknown roles that we included in our study could provide a starting point towards assessing poorly defined mechanisms of resistance against broomrape and discovering novel ones. To date, research on broomrape resistance is mainly focused on the mechanism of SL accumulation in the roots of the host. Our findings suggest that, apart from the unarguable importance of SL metabolism, diverse cellular processes are potentially involved in tomatoes’ responses to broomrapes. Further understanding of such biological processes will expand our knowledge of host resistance to broomrapes and will pave the way to alternative management strategies against parasitism in tomatoes and other crops of high economic and nutritional value.

## 5. Conclusions

Our present research study focused on early-stage infection of tomato with *P. ramosa* and explored the involvement of candidate genes that could serve as molecular markers for future pre-breeding programs. We emphasized exploring further potential mechanisms regarding broomrape resistance by analyzing a large array of genes not previously studied in host–parasite interaction processes and host responses. We investigated herein, for the first time, early-stage broomrape parasitism of two resistant tomato lines, IL6-2 and IL6-3, as compared to the susceptible commercial variety ‘Formula’ and unveiled a series of genes that show remarkable differential expression between resistant and susceptible genotypes upon broomrape infection. Meticulous analysis of gene expression patterns and PEA datasets combined with the mining of bibliographical data has highlighted the potential involvement of the encoded proteins in crucial biochemical pathways implicated in parasitism and resistant mechanisms. Our studies will be expanded to investigate molecular responses to infection along a post-infection time course including additional timepoints. The knowledge to be acquired together with the valuable findings from this study on the genes described above will be used to enrich our understanding of broomrape parasitism at the molecular level and contribute to the development of sustainable solutions for counteracting the devastating effects of broomrape infection in tomatoes and other important crops.

## Figures and Tables

**Table 1:** Gene descriptions, IDs according to NCBI and SolGenomics databases, and gene names used in this manuscript.

NCBI Gene Description	Gene Names in This Manuscript	GeneIDs	SolycIDS
*omega-3 fatty acid desaturase, chloroplastic (FAD)*	*OFA*	LOC107023114	Solyc06g051400
*transcription factor bHLH35 (bHLH)*	*BHLH35*	LOC107024383	Solyc07g018010
*1-phosphatidylinositol-3-phosphate 5-kinase FAB1B-like (FAB)*	*FAB1B*	LOC101246905	Solyc03g123570
*F-box protein At1g78280 (F-box)*	*F-BOX*	LOC107017080	Solyc04g074490
*glutamate decarboxylase 4 (* *GAD)*	*GLDE*	LOC107016348	Solyc04g025530
*MLO-like protein 9*	*MLO*	LOC101254181	Solyc02g038806
*phospholipase D gamma 1-like (PLD)*	*PPDG*	LOC107007945	Solyc01g091910
*zinc finger CCCH domain-containing protein 32 (ZFP)*	*ZINC*	LOC101250699	Solyc06g008740
*CaM2 calmodulin 2*	*CaM2*	SlCaM2	Solyc10g081170
*ATP-citrate synthase beta chain protein 2-like*	*ACLA2*	LOC107007996	Solyc01g059880
*alpha/beta-hydrolase DWARF14-like*	*D14*	LOC101259838	Solyc02g092770
*GABA-TP3 gamma-aminobutyrate transaminase subunit precursor isozyme 3*	*GABA*	GABA-TP3	Solyc12g006450
*phytoene synthase 1*	*PSY*	LOC107014634	Solyc03g031860
*zinc finger protein ZPR1-like*	*ZPR1*	LOC101251441	Solyc02g069120

**Table 2:** Here, 4 out of 14 DEGs studied expressed in annotated pathways in KEGG database.

Gene Name	NCBI ID	SolycID	Pathway Name	Species	Significant Pathway Found
*FAB1B*	LOC101246905	Solyc03g123570	Inositol phosphate metabolism	*S. lycopersicum*	-
*GLDE*	LOC107016348	Solyc04g025530	Alanine, aspartate, and glutamate metabolism	*S. pennelli*	IL6-2_penn, IL6-3_penn
*PPDG*	LOC107007945	Solyc01g091910	Glycerophospholipid metabolism	*S. pennelli*	IL6-2_merged, IL6-3_merged
*CaM2*	SlCaM2	Solyc10g081170	MAPK signaling pathway–plant	*S. lycopersicum*	IL6-2_merged, IL6-3_merged

## Data Availability

The original contributions presented in the study are included in the article and Supplementary Materials. Additionally, the RNA-Seq data reported in this paper have been deposited in the Gene Expression Omnibus (GEO) database under the accession number GSE245472.

## Supplementary Materials

The following supporting information can be downloaded at https://www.mdpi.com/article/10.3390/cimb46080535/s1. Supplementary File S1: Table S1: Tomato plant height (cm) on four different days after inoculation (DAI) with broomrape seeds; Table S2: Tomato plant weight (kg) on four different days after inoculation (DAI) with broomrape seeds; Table S3: Soil plant analysis development (SPAD) for the tomato plants on four different days after inoculation (DAI) with broomrape seeds; Table S4: Relative Water Content (RWC) of tomato leaves on 30 DAI; Table S5: Genomic/transcriptomic alignment rates for *S. lycopersicum* libraries; Table S6: Genomic/transcriptomic alignment rates for introgression line libraries; Table S7: List of the 14 DEGs selected for q PCR validation and information extracted after bioinformatic analysis; Table S8: Primers used for qPCR validation of 14 DEGs; Figure S1: Relative gene expression for the genes A: *ACLA2*, B: *D14*, C: *GABA*, D: *PSY* and E: *ZRP1*, with no significant differences; Figure S2: Genes within each shared pathway for the two species (*S. lycopersicum* X *S. pennellii*) were merged into a single set. The top 20 enriched pathways for each IL (6-2 & 6-3 respectively) are presented; Supplementary File S2: The DEGs, displayed both up- and down-regulation across the different genotypes.
