# Supplementary material for: Transcriptomic Approach for Investigation of Solanum spp. Resistance upon Early-Stage Broomrape Parasitism
Source: Curr Issues Mol Biol. 2024 Aug 18;46(8):9047–73. doi: 10.3390/cimb46080535 (PMC11353193; doi:10.3390/cimb46080535)
Supplement: Supplementary file 1 [file cimb-46-00535-s001.zip › Supplementary_file_1.pdf]

Supplementary File 1

Table S1. Tomato plant height (cm) in four different days after inoculation (DAI) with broomrape seeds.

Tomato height (cm)								
Genotype	0 DAI		15DAI		30DAI		60 DAI	
	control	inoculated	control	inoculated	control	inoculated	control	inoculated
Formula	23.5 ^{a*} ±2.53	26.1^{a} ±2.48	25.5^{b} ±2.06	29.9 ^{a} ±1.76	24.8 ^{b} ±2.23	32.7^{a} ±2.07	26.3 ^{b} ±2.35	31.3^{a} ±1.79
IL 6-2	15.3^{b} ±0.94	13.9 ^{b} ±1.02	16.9^{cd} ±0.72	14.5^{d} ±0.73	16.9^{d} ±0.60	16.8 ^{d} ±0.64	17.1^{cd} ±0.63	15.3 ^{d} ±0.78
IL6-3	17.4 ^{b} ±1.55	16.6 ^{b} ±1.66	18.2 ^{cd} ±1.09	20.4^{c} ±0.85	19.5^{cd} ±1.37	21.6 ^{c} ±1.21	19.3^{cd} ±1.48	21.0 ^{c} ±1.28
Prob> F	<0.001		<0.001		<0.001		<0.001	

* The cell values represent the mean of the varieties per treatment along with the standard error. Means denoted by a different letter indicate significant differences.

Table S2. Tomato plant weight (kg) in four different days after inoculation (DAI) with broomrape seeds.

Tomato weight (kg)								
Genotype	0 DAI		15 DAI		30 DAI		60 DAI	
	control	inoculated	control	inoculated	control	inoculated	control	inoculated
Formula	0.15 ±0,010	0.16 ±0,010	0.17 ±0,012	0.18 ±0,011	0.29 ±0,097	0.19 ±0,010	0.20 ±0,013	0.22 ±0,013
IL 6-2	0.17 ±0,015	0.16 ±0,007	0.18 ±0,010	0.17 ±0,007	0.19 ±0,010	0.18 ±0,008	0.20 ±0,012	0.19 ±0,008
IL 6-3	0,19 ±0,011	0.19 ±0,013	0.20 ±0,011	0.19 ±0,011	0.20 ±0,009	0.21 ±0,011	0.21 ±0,010	0.22 ±0,012
Prob> F	0.14		0.27		0.48		0.44	

* The cell values represent the mean of the varieties per treatment along with the standard error.

Table S3. Soil plant analysis development (SPAD) for the tomato plants in four different days after inoculation (DAI) with broomrape seeds.

SPAD								
Genotype	0 DAI		15DAI		30 DAI		60 DAI	
	control	inoculated	control	inoculated	control	inoculated	control	inoculated
Formula	32.92 ±0,74	30.49 ±1,48	30.99 ±1,27	31.05 ±0,46	31.82d ±1,04	32.58cd ±0,73	30.71 ±1,34	32.68 ±0,71
IL 6-2	31.71 ±1,07	32.77 ±1,52	33.96 ±0,65	32.73 ±1,00	34.94ab ±0,63	35.28a ±0,61	33.2 ±0,72	33.58 ±0,72
IL6-3	29.21 ±2,03	33.59 ±0,51	31.67 ±0,43	32.83 ±0,57	32.94bcd ±0,80	34.18abc ±0,62	33.3 ±0,65	33.65 ±0,93
Prob>F	0.1848		0.0710		0.0102		0.1793	

* The cell values represent the mean of the varieties per treatment along with the standard error. Means denoted by a different letter indicate significant differences.

Table S4. Relative Water Content (RWC) of tomato leaves on 30 DAI (Prod>F 0.5751)

	RWC	
Genotype	control	inoculated
Formula	64.42 ±3,57	69.16 ±4,44
IL 6-2	70.50 ±6,04	63.87 ±1,67
IL6-3	61.40 ±3,60	69.80 ±3,56

Table S5. Genomic/transcriptomic alignment rates for *S. lycopersicum* libraries.

Sample ID	Processedreads	Totalmappedreads	Uniquelymapped	Multi-mapped	Mappingrate (%)	Transcriptome-mappedreads
Fc1	15212589	14229363	14026906	202457	93.5	11857705
Fc2	15400281	14506971	14314548	192423	94.2	12086759
Fc3	14899580	14060243	13885945	174298	94.4	11761498
Fp1	14381851	13457552	13258891	198661	93.6	11256608
Fp2	14311831	12536799	12360811	175988	87.6	10487957
Fp3	10514040	9924140	9771721	152419	94.4	8320014

Table S6. Genomic/transcriptomic alignment rates for introgression line libraries.

Sample ID	Processedreads	Totalmappedreads	Uniquelymapped	Multi-mapped	Mappingrate (%)	Transcr.-mappedreads	S. lyc.-mapped (%)	S. penn.-mapped (%)	Ambig.-mapped (%)
IL6-2c1	15130205	14324301	12863083	1461218	94.7	11980313	87.7	3.5	8.8
IL6-2c2	13075746	12148925	10924818	1224107	92.9	10222260	87.7	4	8.3
IL6-2c3	14066894	13498059	12113054	1385005	96	11466164	87.5	4.3	8.1
IL6-2p2	12351438	11245036	10025167	1219869	91	9514020	86.6	5.8	7.5
IL6-2p3	14130864	13483295	12067356	1415939	95.4	11373586	87.1	5	7.8
IL6-2p5	15256253	14223487	12219123	2004364	93.2	12075176	82.9	9.5	7.5
IL6-3c1	14769400	13959301	12636270	1323031	94.5	11772041	85.2	6.6	8.1
IL6-3c2	13251546	12590892	11116971	1473921	95	10715435	82.8	9	8.1
IL6-3c4	13820401	12706869	11484683	1222186	91.9	10764471	85.2	6.5	8.3
IL6-3p1	14555710	13836304	12482499	1353805	95.1	11647045	84.6	7.2	8.1
IL6-3p2	13886241	13234717	11885438	1349279	95.3	11157135	84.2	7.7	8.1
IL6-3p3	10367191	9447792	8440223	1007569	91.1	8025882	83.6	8.4	7.8

Table S7. List of the 14 DEGs selected for q PCR validation and their data after bioinformatic analysis.

edgeR	Genename	Gene_id	logFC	logCPM	F	PValue	FDR
**Formula_ *S. lycopersicum***	MLO	LOC101254181	0,6124264	4,440851	14,2762239	0,005831302	0,050191342
	ZINC	LOC101250699	-0,9090099	3,350489	14,27595295	0,005831593	0,050191342
	FAB1B	LOC101246905	0,9433622	8,5144902	14,24400141	0,005865969	0,050325395
	CaM2	SlCaM2	0,932536	9,9970355	10,17609065	0,013524197	0,080598879
**IL6-2 _*S.lycopersicum***	MLO	LOC101254181	0,6124264	4,440851	14,2762239	0,005831302	0,050191342
	ZINC	LOC101250699	-0,9090099	3,350489	14,27595295	0,005831593	0,050191342
	FAB1B	LOC101246905	0,9433622	8,5144902	14,24400141	0,005865969	0,050325395
**IL6-2 _*S.pennellii***	GLDE	LOC107016348	-3,9250331	8,0233572	283,6785885	6,12E-11	4,88E-07
	FBOX	LOC107017080	-3,2933072	9,4127743	251,5857689	1,40E-10	5,59E-07
	PPDG	LOC107007945	-3,2644545	6,9587665	212,6424372	4,46E-10	7,73E-07

	OFA	LOC107023114	-3,6945588	10,039286	188,3033829	1,07E-09	9,49E-07
	BHLH35	LOC107024383	-3,840076	7,4965754	168,5778915	2,26E-09	1,21E-06
	ACLA	LOC107007996	-1,1674172	7,3723163	23,72440797	0,000222538	0,003508603
	PSY	LOC107014634	1,3810996	3,7301584	9,662266759	0,007418973	0,042897501
**IL6-3 _*S.lycopersicum***	FAB1B	LOC101246905	2,8825849	8,2260505	87,33315819	2,11E-05	0,00089022
	MLO	LOC101254181	1,9197102	4,2223081	60,69068803	7,40E-05	0,001706932
**IL6-3 _*S.pennellii***	OFA	LOC107023114	-4,8703504	10,195469	451,2810787	4,954E-11	2,1376E-07
	BHLH35	LOC107024383	-5,9196338	7,6628903	416,4785084	7,99503E-11	2,1376E-07
	FBOX	LOC107017080	-3,8801341	10,869603	210,6007753	4,43733E-09	2,62436E-06
	GLDE	LOC107016348	-4,0536538	8,5822507	206,6669237	4,95057E-09	2,62436E-06
	PPDG	LOC107007945	-4,3106816	6,8493516	201,6607965	5,70691E-09	2,62436E-06
	PSY	LOC107014634	0,8284935	4,1208931	4,894414593	0,046666759	0,133160467

Table S8. Primers used for qPCR validation of 14 DEGs. ACT was used as a reference.

Gene name	*NCBI id*	*Forward primer (primer sequence 5′–3′)*	*Reverse primer (primer sequence 5′–3′)*	*Amplicon length(bp)*
ACT	TC194780 (Løvdal, T., & Lillo, C. 2009)[a]	GAAATAGCATAAGATGGCAGACG	ATACCCACCATCACACCAGTAT	159
OFA	LOC107023114	TTGCCGGAGAAGAAGAGGAA	CCAGCCCAAACACAATAGCT	180
BHLH35	LOC107024383	TGTTCTTGACTATTTCGATGAGCC	TGGGACCACAGCTCTAAGTG	157
FAB1B	LOC101246905	GCCTTAAATGTCCACGCGAT	AATGACCACAGTTTGCCACC	153
F-BOX	LOC107017080	CCCAATTGCAAATGCGACCA	ATCCATAGGGGCTCCTCGTT	201
GLDE	LOC107016348	ACCCTGTTACCACTGAGCTT	GCGTTTGTTTTGCCAGTTCC	167
MLO	LOC101254181	TTTTGGCGGTCCTTCATGTG	TTGTTCCCAGCATTTCCAGC	86
PPDG	LOC107007945	ATGGTTACCCTTCGGCATCA	GGCGATGGAGAGAGTCATGA	198
ZINC	LOC101250699	TCATCCTGACCATGCTGAGG	CCTGGGTGAAGCAAAACTGG	186
CaM2	SlCaM2	TTGATCCGGTATAAAAGCAGCA	AGATCTGGTCATCCGTCAGC	161
ACLA2	LOC107007996	GGCGTACCCATGTCATCCAT	CACCAGAGACACAAGGACCA	154
D14	LOC101259838	GCAAGGGACCACTCTGTACC	TACCCGCTTCAACAGGATCG	183
GABA	GABA-TP3	CGACATTCTTTTCATCGCGG	TGCTACAGAGACAAGATCAGGT	131
PSY	LOC107014634	AGAAGAAGGGCTATCTGGGC	ATCGAGCATGTCAAATGGCC	155
ZPR1	LOC101251441	GCTCCATCTCCTGATCCCTC	TCTTGCTCCAACTGATCCGT	186

[a]Corresponding to J.S. Coker etal.,2003, however, note that the original TGI TC Accession numbers have been split and renumbered by the DFCI TGI.

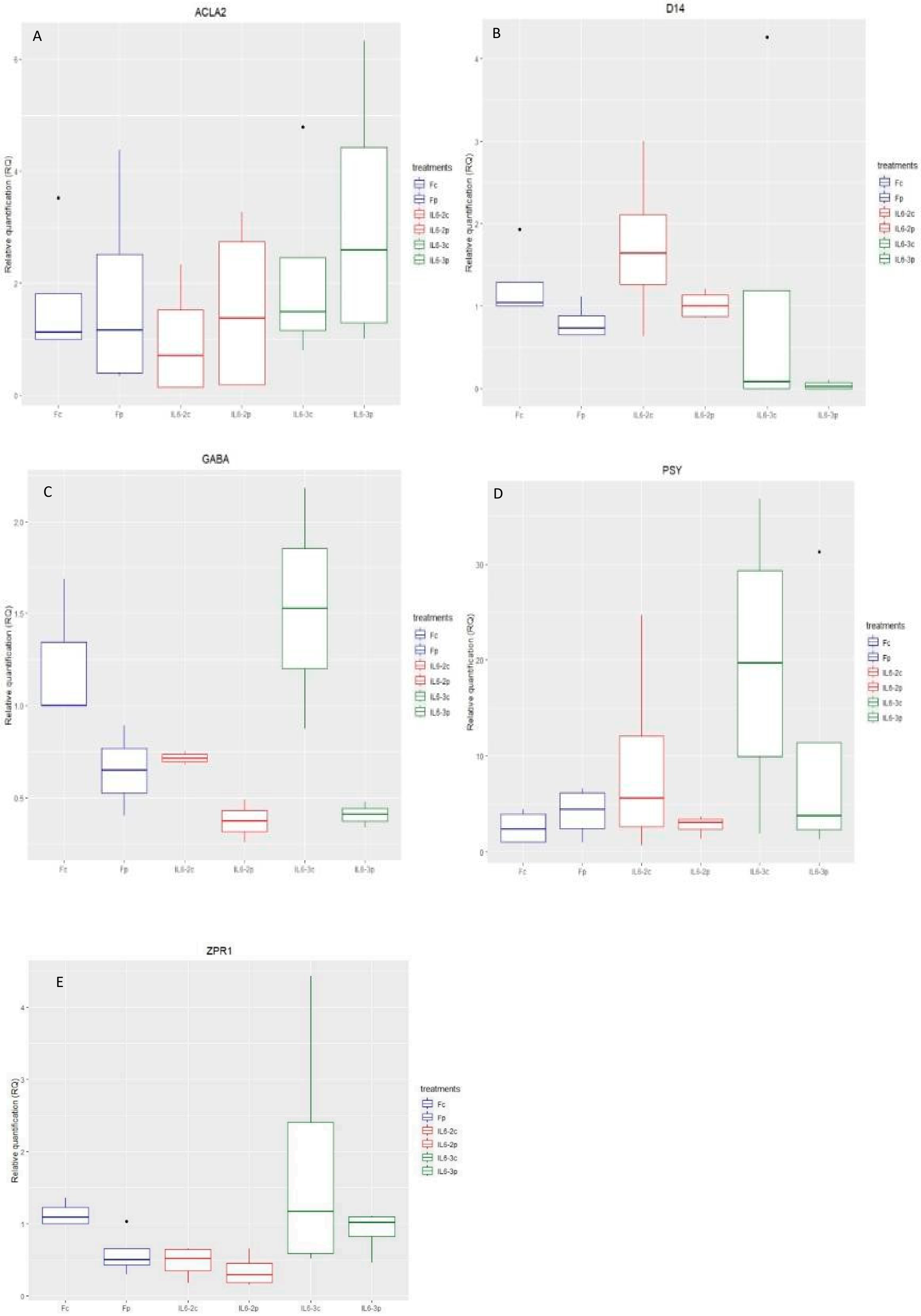

Figure S1. No significant differences in the relative gene expression was found for the genes A: *ACLA2*, *B:D14*, *C:GABA*, *D: PSY* and *E: ZRP1*. Treatments:

Formula control (Fc), Formula Parasitized (Fp), Introgression Line 6-2 control & parasitized (IL 6-2c & IL6-2p respectively), Introgression Line 6-3 control & parasitized (IL 6-3c & IL6-3p respectively). Statistically significant differences were determined for p.value> 0.05.

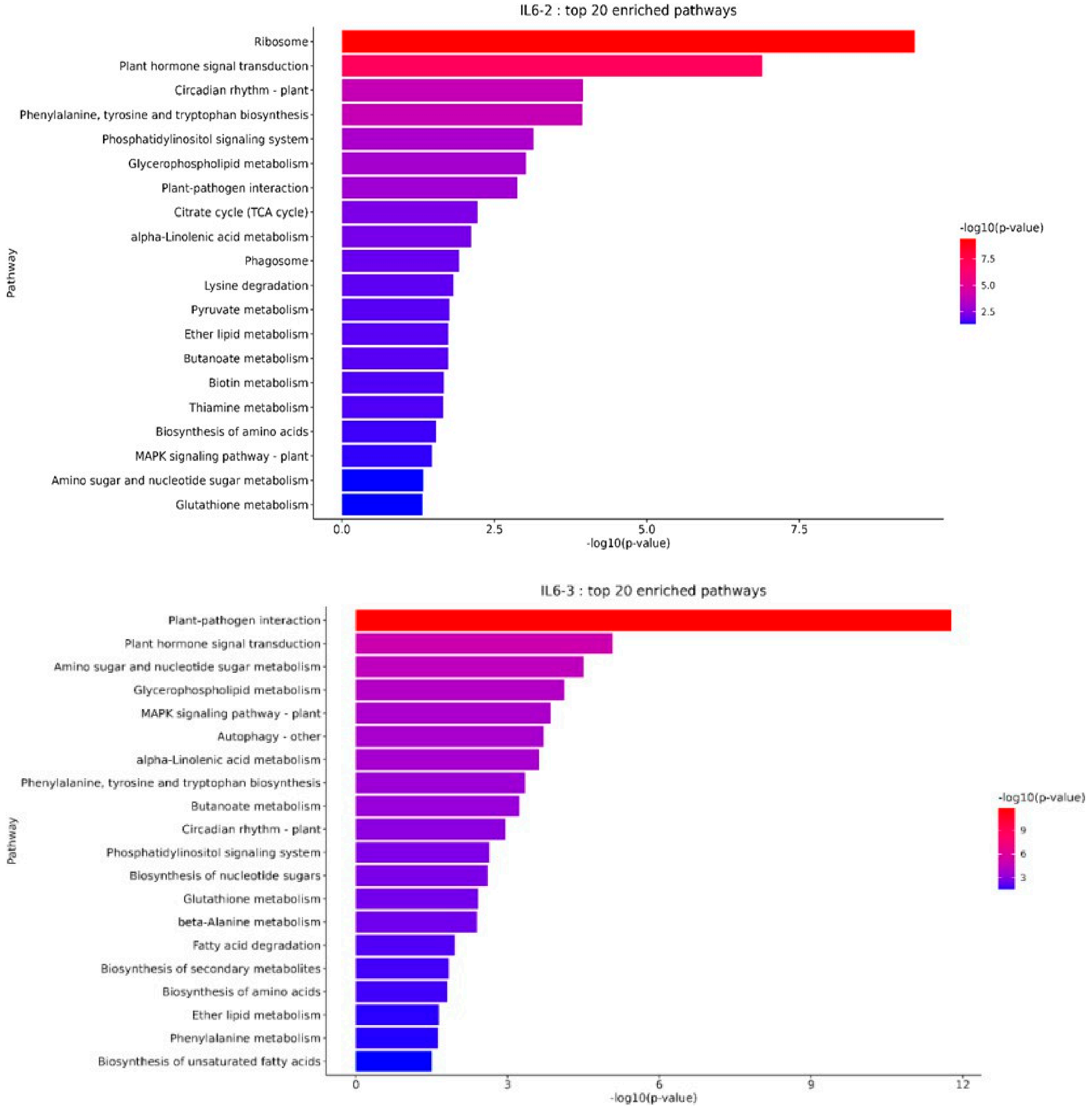

Figure S2. Genes within each shared pathway by the two species (*S.lycopersicum* X *S.pennellii*) were merged into a single set. The top 20 enriched pathways for each IL(6-2 & 6-3 respectively) are presented.
